# Supplementary material for: A Critical View of the Application of the APEX Software (Aqueous Photochemistry of Environmentally-Occurring Xenobiotics) to Predict Photoreaction Kinetics in Surface Freshwaters
Source: Molecules. 2019 Dec 18;25(1):9. doi: 10.3390/molecules25010009 (PMC7017383; doi:10.3390/molecules25010009)
Supplement: Supplementary file 1 [file molecules-25-00009-s001.zip › 3.2.4_gcc-4.4.0.zip!/3.2.4_gcc-4.4.0/3.2.4_gcc-4.4.0/doc/octave/html/interpreter/Comparison-Ops.html]

Comparison Ops - Untitled


Next: Boolean Expressions,
Previous: Arithmetic Ops,
Up: Expressions


---

### 8.4 Comparison Operators

Comparison operators compare numeric values for relationships
such as equality. They are written using
*relational operators*.

All of Octave's comparison operators return a value of 1 if the
comparison is true, or 0 if it is false. For matrix values, they all
work on an element-by-element basis. For example,

```
     [1, 2; 3, 4] == [1, 3; 2, 4]
          =>  1  0
              0  1
```

If one operand is a scalar and the other is a matrix, the scalar is
compared to each element of the matrix in turn, and the result is the
same size as the matrix.

x `<` y: True if x is less than y. x `<=` y: True if x is less than or equal to y. x `==` y: True if x is equal to y. x `>=` y: True if x is greater than or equal to y. x `>` y: True if x is greater than y. x `!=` y x `~=` y: True if x is not equal to y.

String comparisons may also be performed with the `strcmp`
function, not with the comparison operators listed above.
See Strings.

— Function File: **isequal** (x1, x2, ...)  
> Return true if all of x1, x2, ... are equal.
>
>
>
> **See also:** isequalwithequalnans.

— Function File: **isequalwithequalnans** (x1, x2, ...)  
> Assuming NaN == NaN, return true if all of x1, x2, ...
> are equal.
>
>
>
> **See also:** isequal.
